# Predicting Shunt-Dependency After Aneurysmal Subarachnoid Hemorrhage: A Multicenter Validation Study

**DOI:** 10.3390/jcm14238585

**Published:** 2025-12-03

**Authors:** Maryam Said, Christoph Wipplinger, Andrea Cattaneo, Tamara M. Wipplinger, Ekkehard Kunze, Patrick Dömer, Simeon Helgers, Ramazan Jabbarli, Johannes Woitzik

**Affiliations:** 1 Department of Neurosurgery, Evangelisches Krankenhaus Oldenburg, Carl von Ossietzky University Oldenburg, 26129 Oldenburg, Germany; 2 Research Center Neurosensory Science, Carl von Ossietzky University Oldenburg, 26129 Oldenburg, Germany; 3 Department of Neurosurgery, Universitätsklinikum Würzburg, 97080 Würzburg, Germany; 4 Department of Neurosurgery, Cleveland Clinic Florida, Weston, FL 33331, USA; 5 Department of Neurosurgery and Spine Surgery, University Hospital of Essen, 45147 Essen, Germany; 6 Center for Translational Neuro- & Behavioral Sciences (C-TNBS), University Duisburg Essen, 45147 Essen, Germany

**Keywords:** hydrocephalus, prediction, shunt scores, subarachnoid hemorrhage

## Abstract

**Background**: The clinical utility of risk scores predicting shunt dependency after aneurysmal subarachnoid hemorrhage (aSAH) remains limited due to scarce validation data. This multicenter pooled analysis aimed to assess the predictive accuracy of existing post-aSAH shunt risk scores. **Methods**: Consecutive aSAH cases treated at two German university hospitals from January 2010 to July 2023 were pooled into a validation cohort. Total scores for the CHESS, CHESS-Huckman, and SDASH risk models were calculated, and their diagnostic performance was compared using receiver operating characteristic (ROC) curve analysis. **Results**: A total of 813 patients were included, of whom 215 (26.4%) required ventriculoperitoneal shunt placement within a median time of 29 days post-aSAH. All three risk scores were significantly associated with shunt dependency. ROC analysis showed that the CHESS-Huckman score had the highest predictive accuracy (AUC: 0.792, 95% CI: 0.761–0.824), followed by the SDASH (AUC: 0.782, 95% CI: 0.750–0.814) and CHESS (AUC: 0.780, 95% CI: 0.748–0.812) scores. Pairwise comparisons of AUCs were not statistically significant. All three scores showed good overall calibration, with CHESS–Huckman performing best, as confirmed by calibration intercepts and slopes, Brier scores, and decile-based analysis. Higher CHESS–Huckman scores correlated with earlier shunt placement, whereas delayed shunting (>30 days after aSAH) was most common in patients with moderate CHESS–Huckman scores (7–8 points), occurring in 47.4% of cases compared to 41.4% and 33.3% in patients scoring 0–6 and 9–10 points, respectively. **Conclusions**: This multicenter analysis validated existing risk scores for predicting shunt dependency after aSAH, with the CHESS–Huckman score demonstrating the nominally highest diagnostic accuracy. Integrating these risk scores into clinical practice could enhance early identification of patients requiring shunting, potentially reducing external ventricular drain weaning time, shortening hospital stays, and lowering the risk of cerebrospinal fluid infections.

## 1. Introduction

Hydrocephalus after aneurysmal subarachnoid hemorrhage (aSAH) is a frequently occurring and debilitating complication [1]. High incidences have been reported for acute hydrocephalus and up to a third of these patients require permanent cerebrospinal fluid (CSF) diversion for chronic hydrocephalus [2]. While it can be lifesaving, the implantation of a ventriculoperitoneal (VP) shunt carries the risk of complication such as infections, shunt malfunction, and mechanical failure, as well as the burden of lifelong medical surveillance.

The development of hydrocephalus after aSAH is a complex process that may result from a combination of factors, including blood clot formation, inflammation and disturbed CSF circulation and absorption [3,4]. Acute hydrocephalus typically develops within days after ictus, whereas chronic hydrocephalus may manifest weeks or months later. Of note, the incidence of shunt dependency is relatively high in these patients, making it a critical concern in daily clinical practice [2,5].

In recent years, several risk scores have been developed to predict shunt dependency after aSAH, including the Shunt Dependency in SAH (SDASH) score, Chronic Hydrocephalus Ensuing for SAH Score (CHESS) and the CHESS–Huckman score [2,6,7]. These scores were developed to help identify patients at greater risk of developing chronic hydrocephalus and requiring permanent CSF diversion, providing valuable prognostic information. However, the clinical utility of these tools remains limited due to the lack of external validation. While these risk scores show promise, their implementation in daily clinical routine remains wanting.

In this multicenter pooled analysis, we aimed to evaluate the predictive accuracy of existing post-aSAH shunt risk scores using data from two long-term observational cohorts of aSAH patients. By analyzing them, we hope to clarify the utility of the risk scores in predicting the need for shunt placement and dependence, with the goal of improving the care for these severely burdened young patient populations. Once validated, these tools could significantly aid daily practice, allowing for better risk stratification, more timely interventions and improved decision-making in the management of hydrocephalus following aSAH. We aim to enhance and contribute to the development of more reliable, evidence-based approaches in the management of aSAH patients.

## 2. Materials and Methods

### 2.1. Patient Population

In this large retrospective study, the data of all consecutive aSAH cases treated at two German university hospitals between January 2010 and July 2023 with high treatment volumes were analyzed. All adult patients (>18 years) with available pre-treatment computed tomography (CT) scans performed within 48 h after ictus, allowing the assessment of the radiographic severity of aSAH and the measurement of the ventricular system were included. Subjects were excluded if they did not receive aneurysm treatment, were transferred to another hospital or died within 14 days before successful completion of external ventricular drain (EVD) weaning and without undergoing shunt placement, or if an admission CT scan prior to EVD placement was unavailable.

### 2.2. aSAH Management

In both university hospitals, the standard of care for ruptured intracranial aneurysms was similar. After initial diagnosis of the aSAH in the native CT scan, which was mostly followed by a CT-angiography, diagnostics were completed with a digital subtraction angiography of the intracranial vessels. All aSAH patients were admitted to the intensive care unit for monitoring. Treatment of the ruptured aneurysm usually followed within 24 h, either by endovascular treatment or microsurgical intervention. Patients received a standardized baseline therapy (i.e., maintenance of normovolemia, mean arterial pressure > 70 mmHg—adapted for vasospasms—and oral nimodipine and/or magnesium) for the first 14 (Cohort A) or 21 days (Cohort B) after the initial bleed. EVD placement was indicated for patients with (i) neurological deterioration or depressed level of consciousness consistent with hydrocephalus, accompanied by (ii) radiographic evidence of acute hydrocephalus on CT (ventriculomegaly with or without transependymal CSF flow) and/or obstructive intraventricular hemorrhage. Later, patients were weaned from the drainage if contraindications such as meningitis or persistently increased intracranial pressure (ICP) were not present. In one cohort, the EVD was closed for 48 h (rapid weaning, Cohort A), whereas the drainage was weaned gradually by elevating the threshold of the drainage and performing lumbar puncture or placing a lumbar drainage in the other cohort (gradual weaning, Cohort B). Weaning of the CSF drainage was considered successful if no pathologically increased ICPs (>20 mmHg) occurred during the closing time, patients did not develop new neurological deficits or increasing headache reversible by opening the EVD and the ventricles were not enlarged on the post-weaning CT-scan.

Indications for permanent CSF shunt implantation were standardized and based on a combination of clinical and radiological findings. A shunt was placed in patients with persistent or recurrent clinical signs of hydrocephalus after EVD closure or removal (e.g., deterioration of consciousness, headache, gait disturbance), radiographic evidence of progressive or persistent ventriculomegaly on CT, and/or failure of EVD weaning, defined as recurrence of elevated ICP (>20 mmHg), neurological decline, or need for reopening of the EVD. These criteria were applied consistently in both participating centers.

Additional CT scans were performed before and after the placement of a permanent CSF shunt (commonly ventriculoperitoneal), after any other intracranial surgical procedure, and with neurological deterioration.

### 2.3. Score Calculation

For each patient, the first available CT-scan that was performed after admission and before the start of any treatment was used to measure the Huckman index (i.e., the maximum width between the two frontal horns added to the minimum width of the ventricles between caudate nuclei) [8]. The other parameters of the CHESS–Huckman, CHESS and SDASH risk scores for hydrocephalus were also acquired to calculate the total score values. For the CHESS and the CHESS–Huckman scores, the other score elements included acute hydrocephalus, Hunt & Hess grade 4–5, intraventricular hemorrhage, ruptured aneurysm in the posterior circulation and cerebral infarction within 72 h after SAH. The parameters of the SDASH score included acute hydrocephalus, Hunt & Hess grade 4–5 and Barrow Neurological Institute score [9] 3–5. An overview of the weight of the components of these scores is provided in Supplementary Table S1.

### 2.4. Data Management

All clinical and radiographic parameters used for score calculation were obtained retrospectively from the institutional electronic health records of the participating centers. Most variables were automatically extracted with the support of hospital IT services, ensuring a high level of data accuracy and completeness. Extracted data were then manually reviewed and verified by the study investigators at each site to correct potential inconsistencies or missing values, serving as an internal quality control process. The data were anonymized, centrally reviewed and merged into a single database. The study was conducted in accordance with the Declaration of Helsinki and approved by our institutional ethic committees.

### 2.5. Study Endpoints and Statistical Analyses

This study was conducted to assess the predictive accuracy of several existing post-SAH shunt risk scores in two long-term observational aSAH cohorts utilizing different EVD weaning concepts. Furthermore, the association between the risk score with the highest diagnostic accuracy and the timing of shunt placement was studied. Shunt timing (i.e., time between aneurysm rupture and shunt placement) was analyzed as a continuous variable and in dichotomized manner, where the shunt placement more than 30 days after aSAH was defined as delayed shunting.

Binary logistic regression analyses were performed to evaluate the association between each risk score and shunt dependency, with cohort allocation (i.e., EVD weaning protocol) included as a categorical covariate. The Enter method was used for variable entry in all models.

Model calibration was evaluated using several complementary approaches. First, calibration intercepts and slopes were obtained through logistic recalibration models (logit[p] = α + β × score). Model goodness-of-fit was assessed using the Hosmer–Lemeshow test, with *p*-values > 0.05 indicating adequate fit. Second, overall predictive accuracy was quantified using the Brier score. Third, calibration was further explored through decile-based grouping of predicted probabilities, comparing observed and predicted shunt rates across ten risk strata.

Discrimination was evaluated using the receiver operating characteristic (ROC) curve analysis, and pairwise differences in AUC values were assessed using the DeLong test for correlated ROC curves. Associations between risk score values and shunt placement rates were analyzed using the Chi^2^ or Fisher exact test, as appropriate. Statistical analyses were performed using IBM SPSS Statistics (Version 31.0.1.0, IBM Corp., Armonk, NY, USA), and a two-sided *p*-value ≤ 0.05 was considered statistically significant.

## 3. Results

A total of 961 aSAH patients were treated across both centers in the allocated study period. Hereof, 813 met the eligibility criteria and were included in the final analysis cohort. Among these patients, 215 (26.4%) required a ventriculoperitoneal shunt after aSAH, with a median time of 29 days after hemorrhage (interquartile range [IQR] 23–56 days; range 10–954 days). The shunt rates were similar between the two cohorts (26.9% vs. 26.1%; *p* = 0.809). The baseline study-related characteristics of all patients are presented in Table 1.

### 3.1. Discrimination of Prediction Scores

**Figure 1:**
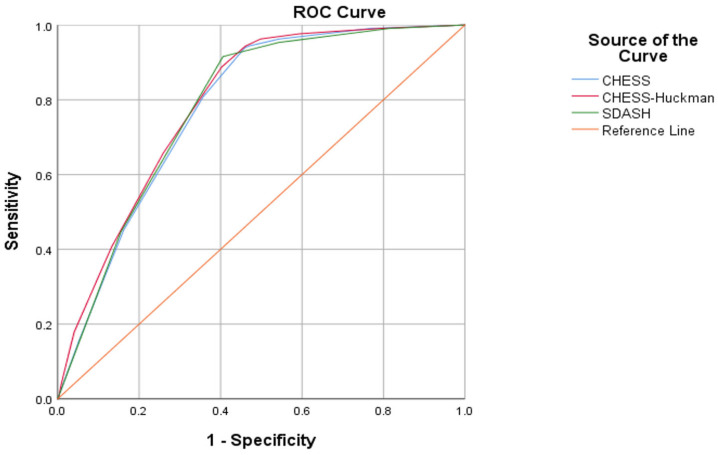
The CHESS, CHESS–Huckman and SDASH risk scores all demonstrate a significant association with shunt dependency. The CHESS–Huckman score performs best with the largest area under the curve (AUC) value in the ROC analysis (0.792; 95% confidence interval [CI]: 0.761–0.824), followed by the SDASH score (0.782; 95% CI: 0.750–0.814) and the CHESS (0.780; 95% CI: 0.748–0.812).

In the logistic regression analysis, we found that regardless of cohort allocation and weaning modality, all three risk scores demonstrated a significant association (*p* < 0.0001) with shunt dependency, as is outlined in Table 2. All three scores showed comparable discrimination. The CHESS–Huckman score proved to have the highest area under the curve (AUC) value in the ROC analysis (0.792; 95% confidence interval [CI]: 0.761–0.824), followed by the SDASH score (0.782; 95% CI: 0.750–0.814) and the CHESS (0.780; 95% CI: 0.748–0.812, Figure 1). Pairwise comparisons of AUCs were not statistically significant (all *p* > 0.05). The CHESS–Huckman score also presented with better precision–recall curve as compared to the other two shunt prediction scores (Supplementary Figure S1). Overall model quality and parameters of the classifier evaluation metrics (Supplementary Table S2) also showed higher predictive values for the CHESS–Huckman score. There was no significant improvement in the AUC values in any paired combinations of these scores (Supplementary Table S3).

**Figure 2:**
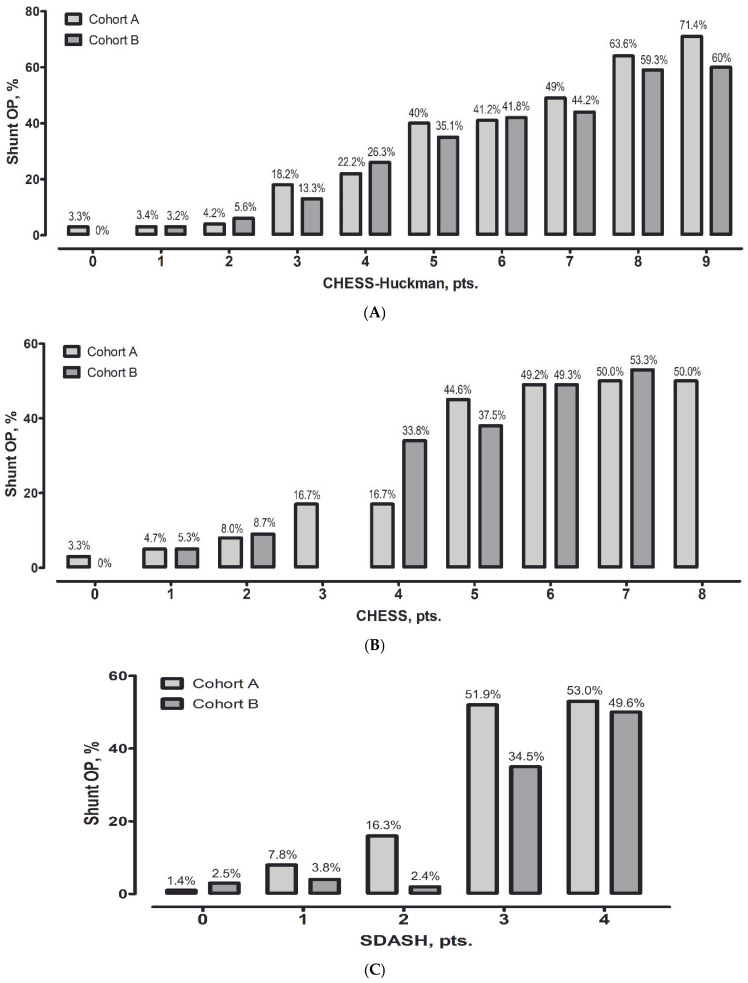
The percentage of permanent cerebrospinal fluid shunts is outlined against the number of points on the CHESS–Huckman risk score for both cohorts. With every point increase, the probability of permanent shunt requirement is increased in both cohorts (**A**). For reference, the performances of the CHESS (**B**) and SDASH (**C**) scores are also depicted.

To further evaluate the impact of different EVD weaning strategies, diagnostic accuracy was analyzed separately within each cohort. All three scores demonstrated good predictive performance in both cohorts, with no statistically significant differences in pairwise comparisons. AUC values were slightly higher in Cohort A (rapid weaning) compared to Cohort B (gradual weaning): CHESS–Huckman 0.807 vs. 0.776; SDASH 0.804 vs. 0.767; and CHESS 0.787 vs. 0.763. Adjustment for cohort allocation did not materially alter the predictive performance of any score. These findings support the robustness of the tested scores across different EVD weaning strategies. Additional analysis showed that, even when considering both cohorts separately, the risk of shunt dependency gradually increased with increasing values of the CHESS–Huckman score in both cohorts (Figure 2).

### 3.2. Calibration of Prediction Scores

In addition to discrimination, calibration was assessed for all three shunt prediction scores (Supplementary Table S4). Logistic recalibration yielded intercepts close to zero and slopes close to one for all three scores, indicating overall good calibration. Hosmer–Lemeshow goodness-of-fit tests were non-significant for CHESS–Huckman (χ^2^ = 9.47, df = 6, *p* = 0.149) and CHESS (χ^2^ = 9.73, df = 5, *p* = 0.083), whereas SDASH showed a significant result (χ^2^ = 11.30, df = 3, *p* = 0.010), suggesting minor deviations from perfect fit at higher risk levels.

Brier scores confirmed these findings, with all values well below the commonly accepted threshold of 0.20 for good predictive performance (CHESS–Huckman: 0.155; CHESS: 0.158; SDASH: 0.158).

Decile-based calibration analysis (Supplementary Table S5 and Supplementary Figure S3) further demonstrated good agreement between predicted and observed probabilities for CHESS and CHESS–Huckman across most of the risk spectrum, with mild overestimation in the highest decile for all three scores. SDASH showed slightly larger discrepancies in the upper risk deciles. Overall, these results indicate robust calibration for CHESS and CHESS–Huckman and acceptable calibration for SDASH.

### 3.3. Prediction of Shunt Timing

**Figure 3:**
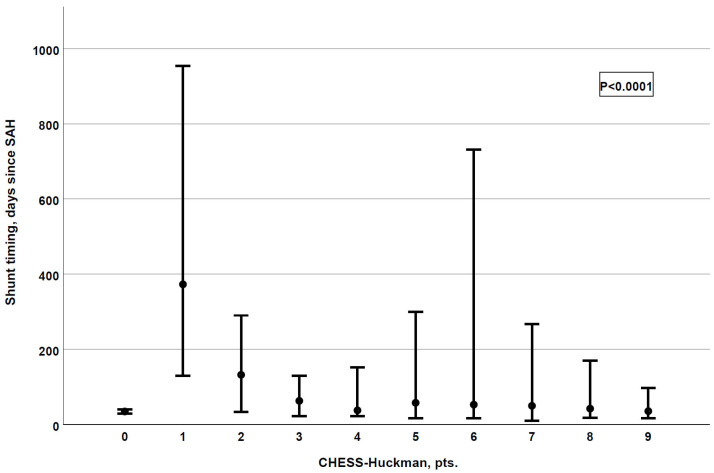
The CHESS–Huckman risk score shows a strong association with shunt timing. The higher the score, the earlier patients receive their permanent shunts (*p* < 0.0001, correlation coefficient: −0.255).

**Figure 4:**
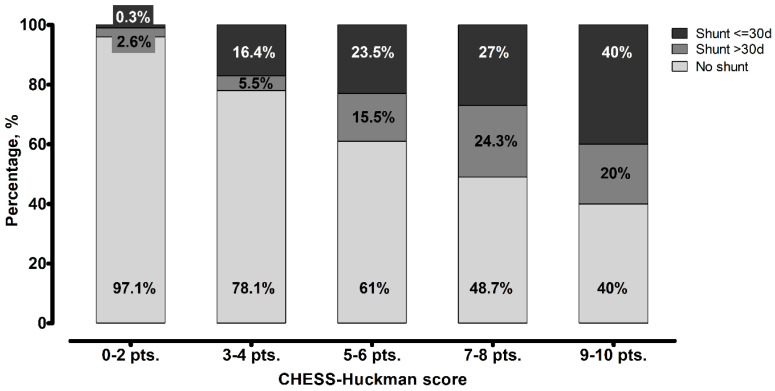
Distribution of patients based on the timing of shunt placement in different scores values of the CHESS–Huckman score. The likelihood of patients requiring a shunt within 30 days after aneurysmal subarachnoid hemorrhage increases with higher scores. Delayed shunt placement was most common among patients with moderate scores (7–8 points).

Finally, we analyzed the association between the CHESS–Huckman score and the shunt timing. Patients with higher scores tended to receive permanent CSF shunts earlier (*p* < 0.0001), as depicted in Figure 3. The results of the association between the CHESS–Huckman score and shunt timing in dichotomized manner (i.e., early vs. delayed shunting) are provided in Figure 4. Of note, the delayed placement of permanent shunts was most common in patients with moderate CHESS–Huckman scores (7–8 points), occurring in 47.4% (37 out of 78) of the shunt-dependent cases. To compare, 41.4% (53 out of 128) of shunted patients with low scores (0–6 points) acquired the shunt in over 30 days after aSAH, and 33.3% (3 out of 9) of patients with high CHESS–Huckman scores (9–10 points).

## 4. Discussion

aSAH remains one of the most devastating neurological emergencies and posthemorrhagic hydrocephalus is one of its most common complications. Hydrocephalus can occur acutely or chronically following an aSAH and both forms are associated with significant morbidity and require timely recognition and management to improve patient outcomes [10,11,12]. In this multicenter study, the existing risk scores for prediction of shunt dependency were successfully validated. When only modest, the CHESS–Huckman score demonstrated the highest diagnostic accuracy.

Hydrocephalus after aSAH is typically caused by an obstruction of CSF flow, often due to the presence of blood or clots in the subarachnoid space, which can obstruct the arachnoid villi or the cerebral aqueduct. Subsequent inflammatory processes can also impair the absorption of CSF and lead to chronic hydrocephalus [3,4,13]. These mechanisms are aggravated by the ICP that accompanies both the hemorrhage and the hydrocephalus, leading to further cerebral injury, especially when the hydrocephalus goes unrecognized.

Several clinical factors have been identified as predictors of the development of hydrocephalus after aSAH, including the severity of the initial hemorrhage, the volume of blood present in the subarachnoid space and the occurrence of delayed cerebral ischemia [14,15,16]. Patients with a higher Hunt and Hess grade at presentation are more likely to develop post-aSAH hydrocephalus, as can be deduced from the risk scores in the present study. Additionally, aneurysm location can increase the risk of CSF obstruction, particularly if the rupture occurs near areas such as the basal cisterns [17,18].

Patients with chronic hydrocephalus often require more extended periods of monitoring, treatment and rehabilitation [19]. While prolonged EVD use can help maintain CSF drainage and prevent the recurrence of increased ICP, it also carries risks, such as infection, catheter obstruction and complications related to prolonged immobility [20]. Longer hospitalization is related with higher risks of secondary complications, including hospital-acquired infections, venous thromboembolism and muscle deconditioning. Occurrence of these hospital-acquired complications is independently associated with sustained poor clinical outcomes 12–18 months after aSAH [21].

Delaying permanent CSF diversion can necessitate prolonged or repeated external ventricular drainage, which may not adequately control hydrocephalus and often result in additional surgical procedures. Such prolonged or repeated interventions are associated with a higher risk of infection and related complications [22,23]. Conversely, CSF infections occurring during EVD weaning can themselves delay or complicate the timing of shunt placement.

One of the most noteworthy findings of the present study is that the probability of delayed shunt placement was highest among patients with moderate CHESS–Huckman scores (7–8 points). This pattern may reflect the fact that patients in this intermediate risk group are more likely to undergo successful initial EVD weaning without the need for immediate shunt insertion compared to those with higher scores. However, due to the still substantial impact of the initial aSAH, these patients appear to remain susceptible to the later development of clinically significant chronic hydrocephalus, ultimately requiring shunt surgery.

From a clinical perspective, this subgroup represents a diagnostically challenging intermediate-risk population: patients are neither low-risk enough to confidently avoid further surveillance nor high-risk enough to justify early shunt implantation. In this setting, delayed symptom onset or a more insidious course of chronic hydrocephalus may lead to postponed intervention. Furthermore, the absence of overt clinical or radiographic failure during initial EVD weaning may encourage prolonged observation, which can shift definitive shunt placement into the subacute or chronic phase.

These findings suggest that patients with moderate CHESS–Huckman scores may particularly benefit from structured follow-up protocols, closer monitoring after EVD removal, or standardized reassessment strategies during rehabilitation. Future studies may help define whether this subgroup could be targeted for earlier or more standardized diagnostic and therapeutic interventions to avoid unnecessary delays in shunt treatment.

Another important consequence of delayed hydrocephalus management is the need for secondary admissions. In our cohort, a substantial portion of patients underwent permanent shunt implantation more than 30 days after ictus. Untreated or inadequately managed hydrocephalus hampers rehabilitation, particularly in patients with cognitive deficits and motor dysfunction and has been associated with poorer long-term function outcomes, including reduced independence in daily activities [24]. Furthermore, early recognition of shunt dependency could avoid secondary admissions and reduce costs. Patients with hydrocephalus after aSAH have been shown to experience significantly longer hospital stays and higher readmission rates compared to those without hydrocephalus, resulting in a considerable health-economic burden [25].

Therefore, one of the most important aspects of managing hydrocephalus after aSAH is the early recognition of patients who are at risk for developing chronic hydrocephalus. Proper and timely risk assessment opens the possibility for more targeted management strategies, such as the use of early shunt placement in high-risk patients. In a study by Kang et al., the early conversion from EVD to a permanent shunt did not result in shunt-related infections and a marginal revision rate, even in the presence of intraventricular hemorrhage and high protein content in the CSF at the time of shunt placement [26].

We believe that a risk-score-driven approach to managing chronic hydrocephalus—promoting early shunt placement in high-risk patients and timely EVD weaning in aSAH individuals with a low-risk profile—may help reduce secondary complications. Additionally, this strategy may contribute to a reduced length of initial hospitalization and a lower rate of readmissions for delayed shunt placement, yielding not only neurological advantages but also socioeconomic benefits.

While our analysis focused on the CHESS, CHESS–Huckman, and SDASH scores due to their established validation and reliance on routinely available clinical and radiographic parameters, several other shunt prediction scores and models have been proposed, such as the PS3 [27], AFA [28], and MAI scores [29], as well as more recent nomogram-based approaches [30]. Many of these tools incorporate additional variables that were not available in our dataset and therefore could not be reliably tested in this study. Nevertheless, their existence underlines the growing interest in early shunt dependency prediction. Further independent validations are needed to determine which of these tools are best suited for clinical routine and to enable evidence-based selection among the increasing number of available scores.

The major limitation of our study is its retrospective design. Several patients had to be excluded because the inclusion criteria were not sufficiently met. Nevertheless, we believe that our findings are still representative of aSAH patients because two large independent cohorts from two different university clinics were included, supporting generalizability. Importantly, external validation of the scores was performed in two distinct EVD weaning modalities, which in our view strengthens the results. As the use of (additional) lumbar drainage was shown to lessen the burden of secondary infarction and unfavorable outcome [31,32,33,34] and therefore the utilization of lumbar drainage in SAH patients might increase in future, the possible impact of lumbar drainage on the diagnostic accuracy of the risk scores such as SDASH, CHESS and CHESS–Huckman needs further clarification. In addition, differences in local indication patterns for EVD placement across neurovascular centers—including the use of prophylactic EVD insertion prior to endovascular aneurysm treatment requiring dual antiplatelet therapy in some institutions—may also influence shunt dependency rates and thus limit the generalizability of our findings to centers with different clinical protocols. Furthermore, as the Huckman index was measured by a single rater at each center, inter-rater variability within individual cohorts was not applicable. Due to data protection and ethical regulations, cross-center inter-rater reliability assessments could not be performed, which represents a methodological limitation of this study. Finally, although patients in whom shunt dependency could not be assessed due to early death or transfer were excluded to avoid classification bias, this may still introduce a degree of selection bias inherent to retrospective cohort studies.

## 5. Conclusions

In our large multicenter analysis, we successfully validated the CHESS–Huckman score as well as the previously researched SDASH and CHESS. Overall, the CHESS–Huckman score performed best in diagnostic accuracy. The standard implementation of aSAH shunt dependency risk scores in daily clinical practice could aid in the early identification of patients who are likely to profit from a permanent shunt placement. This could optimize EVD weaning time, lead to reduced lengths of hospital stay, lower risk of CSF infections and hospital readmission, lower health care costs and increased rehabilitation potential.

## Figures and Tables

**Table 1:** Baseline of included patients’ characteristics.

	Cohort A		Cohort B	
Parameter	Count/Mean	%/SD	Count/Mean	%/SD
Age (years)	56	13	55	12
Sex				
Male	124	36.8%	155	32.6%
Female	213	63.2%	321	67.4%
Initial neurological condition:				
Hunt&Hess Grade I–III	236	70.0%	301	63.2%
Hunt&Hess Grade IV–V	101	30.0%	175	36.8%
Acute hydrocephalus:				
No	157	46.6%	180	37.2%
Yes	180	53.4%	296	62.2%
Aneurysm location:				
Anterior circulation	261	77.7%	349	73.3%
Posterior circulation	75	22.3%	127	26.7%
Treatment modality				
Endovascular	198	59.5%	317	66.7%
Clipping	135	40.5%	158	33.3%
CHESS				
0	61	18.2%	80	16.8%
1	64	19.1%	76	16.0%
2	25	7.5%	23	4.8%
3	6	1.8%	1	0.2%
4	12	3.6%	77	16.2%
5	56	16.7%	136	28.6%
6	65	19.4%	68	14.3%
7	42	12.5%	15	3.2%
8	4	1.2%	0	0.0%
CHESS–Huckman score				
0	60	17.9%	67	14.1%
1	59	17.6%	62	13.0%
2	24	7.2%	36	7.6%
3	11	3.3%	15	3.2%
4	9	2.7%	38	8.0%
5	40	11.9%	94	19.7%
6	51	15.2%	80	16.8%
7	51	15.2%	52	10.9%
8	22	6.6%	27	5.7%
9	7	2.1%	5	1.1%
10	1	0.3%	0	0.0%
SDASH score				
0	73	21.9%	40	8.4%
1	64	19.2%	105	22.1%
2	49	14.7%	41	8.6%
3	81	24.3%	168	35.3%
4	66	19.8%	122	25.6%
Shunt placement				
No shunt	247	73.1%	351	73.9%
Shunt within 30 days	51	15.1%	73	15.4%
Shunt > 30 days	40	11.8%	51	10.7%

Abbreviations: SD = standard deviation, CHESS = Chronic Hydrocephalus Ensuing from SAH Score, SDASH = Shunt Dependency in SAH.

**Table 2:** Binary logistic regression analysis for the associations between risk scores and shunt dependency adjusted for the cohort type.

	Odds Ratio (95% CI)	*p*-Value
Risk Scores (Per-Score-Increase)		
*CHESS*	1.76 (1.58–1.94)	**<0.0001**
*CHESS–Huckman*	1.66 (1.52–1.81)	**<0.0001**
*SDASH*	2.80 (2.33–3.36)	**<0.0001**

Abbreviations: CI = confidence interval, CHESS = Chronic Hydrocephalus Ensuing from SAH (subarachnoid hemorrhage) Score, SDASH= Shunt Dependency in SAH. Significant findings are in bold.

## Data Availability

The data presented in this study are available on request from the corresponding author due to ethical restrictions.

## Supplementary Materials

The following supporting information can be downloaded at: https://www.mdpi.com/article/10.3390/jcm14238585/s1, Table S1: Components and weights of parameters in the combined CHESS-Huckman score; Table S2: Components and weights of parameters in the SDASH score; Table S3: Components and weights of parameters in the CHESS score; Table S4: Classifier evaluation metrics for the CHESS, CHESS-Huckman and SDASH scores for shunt prediction after SAH in the pooled cohort; Table S5: Paired-sample area difference under the ROC curves for the tested shunt prediction scores; Figure S1: Precision-Recall curve showing the higher diagnostic accuracy of the CHESS-Huckman score as compared to the CHESS and SDASH scores for the prediction of shunt dependency in the pooled cohort; Figure S2: Overall model quality of the SDASH, CHESS-Huckman and CHESS scores for the prediction of shunt dependency after SAH in the pooled cohort; Figure S3: Decile-based calibration plot of CHESS, CHESS–Huckman, and SDASH scores for the prediction of shunt dependency after SAH in the pooled cohort.

## Abbreviations

The following abbreviations are used in this manuscript:

aSAH	Aneurysmal subarachnoid hemorrhage
SD	Standard deviation
CHESS	Chronic Hydrocephalus Ensuing from SAH Score
SDASH	Shunt Dependency in SAH
ROC	Receiver operation curve
AUC	Area under the curve
CI	Confidence interval
EVD	External ventricular drainage
VP-Shunt	Ventriculoperitoneal shunt
CT-scan	Computed tomography scan
CSF	Cerebrospinal fluid
ICP	Intracranial pressure
