# Supplementary material for: Predicting Shunt-Dependency After Aneurysmal Subarachnoid Hemorrhage: A Multicenter Validation Study
Source: J Clin Med. 2025 Dec 3;14(23):8585. doi: 10.3390/jcm14238585 (PMC12693472; doi:10.3390/jcm14238585)
Supplement: Supplementary file 1 [file jcm-14-08585-s001.zip › jcm-3949291-supplementary.pdf]

Supplementary Table S1.

Components and weights of parameters in the combined CHESS-Huckman score.

**Parameter**	**Score weight**
Acute hydrocephalus	4
Initial clinical condition (Hunt & Hess grade 4-5)	1
Intraventricular hemorrhage	1
Ruptured aneurysm in the posterior circulation	1
Early cerebral infarction (within 72 hours after SAH)	1
Huckman index ≥6.0 cm	2

Table S2. Components and weights of parameters in the SDASH score.

**Parameter**	**Score weight**
Acute hydrocephalus	2
Hunt & Hess grade ≥ 4	1
Barrow Neurological Institute score ≥ 3*	1

Table S3. Components and weights of parameters in the CHESS score.

**Parameter**	**Score weight**
Acute hydrocephalus	4
Initial clinical condition (Hunt & Hess grade 4-5)	1
Intraventricular hemorrhage	1
Ruptured aneurysm in the posterior circulation	1
Early cerebral infarction (within 72 hours after SAH)	1

* Measuring maximal subarachnoid hemorrhage (SAH) thickness to predict delayed cerebral ischemia (DCI)

Supplementary Table S4: Classifier evaluation metrics for the CHESS, CHESS-Huckman and SDASH scores for shunt prediction after SAH in the pooled cohort

**Score**	**Gini Index**	**K-S Statistics**	
		**Max K-S***	**Cutoff †**
CHESS	0.557	0.478	3.50
CHESS-Huckman	0.583	0.483	4.50
SDASH	0.561	0.508	2.50

* The maximum Kolmogorov-Smirnov (K-S) metric. Also, the maximum value of Youden's index.
† In case of multiple cutoff values associated with Max K-S, the largest one is reported.

Supplementary Table S5: Paired-sample area difference under the ROC curves for the tested shunt prediction scores

**Score**	**Asymptotic**		**AUC Difference**	**Std. Error Difference †**	**Asymptotic 95% Confidence Interval**	
	**z**	**Sig. (2-tail)***			**Lower Bound**	**Upper Bound**
CHESS - CHESSHuckman	-1.397	0.162	-0.013	0.177	-0.032	0.005
CHESS - SDASH	-0.196	0.845	-0.002	0.177	-0.024	0.020
CHESSHuckman - SDASH	0.907	0.365	0.011	0.176	-0.013	0.035

* Null hypothesis: true area difference = 0

† Under the nonparametric assumption

Supplementary Figure S1: Precision-Recall curve showing the higher diagnostic accuracy of the CHESS-Huckman score as compared to the CHESS and SDASH scores for the prediction of shunt dependency in the pooled cohort.

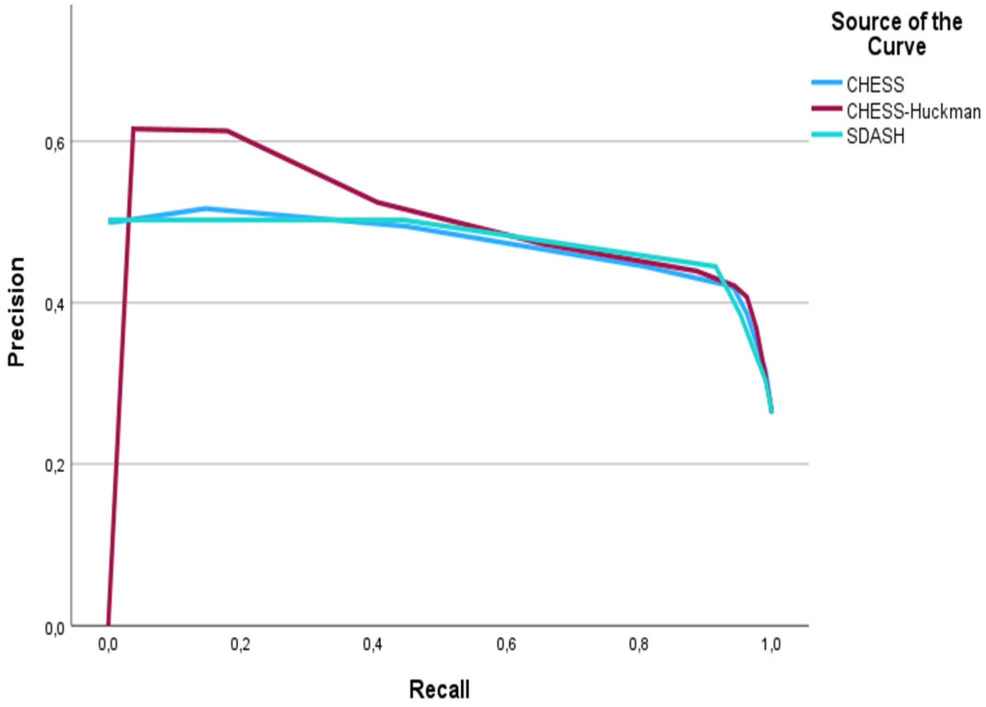

Supplementary Figure S2: Overall model quality of the SDASH, CHESS-Huckman and CHESS scores for the prediction of shunt dependency after SAH in the pooled cohort.

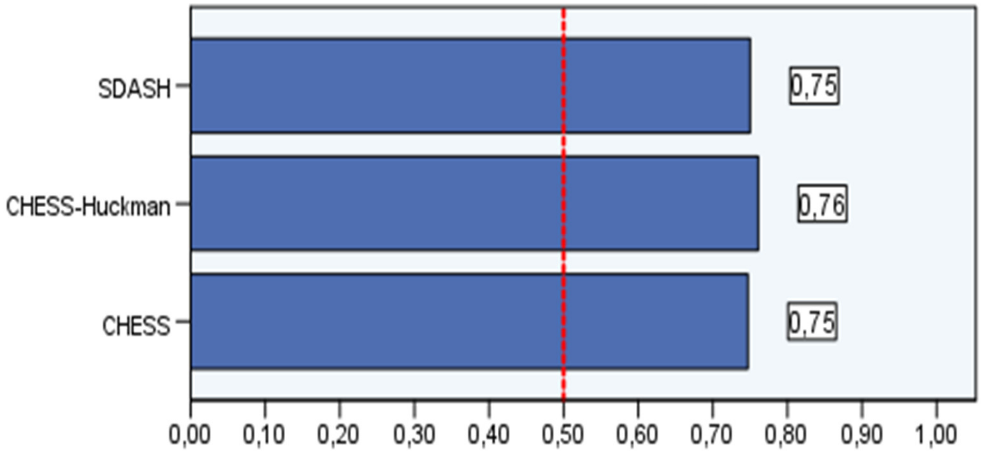

Supplementary Figure S3: Decile-based calibration plot of CHESS, CHESS-Huckman, and SDASH scores for the prediction of shunt dependency after SAH in the pooled cohort.

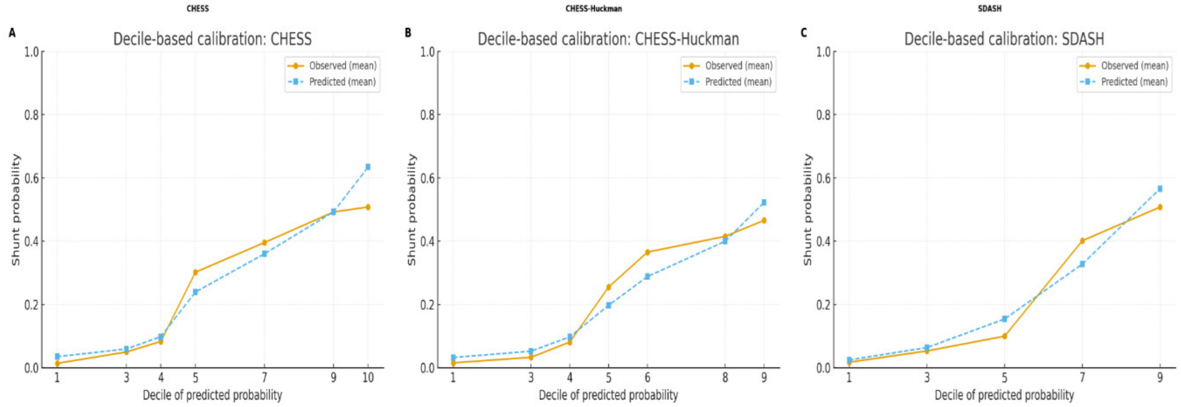

Supplementary Figure S3. Decile-based calibration plots for CHESS (A), CHESS-Huckman (B), and SDASH (C).
